# Clinical and pathological study of mutL homolog 1-deficient gastric cancer and the search for background gene expression changes

**DOI:** 10.1007/s00595-026-03279-z

**Published:** 2026-04-07

**Authors:** Nobufumi Sekino, Yoshihiro Kurata, Koichi Hayano, Yasunori Matsumoto, Ryota Otsuka, Sho Hirano, Masaya Uesato, Takeshi Toyozumi, Akira Nakano, Tadashi Shiraishi, Keisuke Matsusaka, Jun-ichiro Ikeda, Hideki Hayashi, Hisahiro Matsubara

**Affiliations:** 1 Department of Frontier Surgery, Graduate School of Medicine, Chiba University, Chiba, Japan; 2 Division of Laboratory Medicine, Chiba University Hospital, Chiba, Japan; 3 Department of Diagnostic Pathology, Graduate School of Medicine, Chiba University, Chiba, Japan

**Keywords:** Gastric adenocarcinoma, MLH1 defi ciency, Microsatellite instability, PD-L1, NF-kappaB

## Abstract

**Purpose:**

Microsatellite instability caused by impaired mismatch repair (MMR) is referred to as MSI-high, and gastric cancers with this feature are classified as MSI-high gastric cancer. The loss-of-function of mutL homolog 1 (MLH1) is a major cause of MSI-high gastric cancer. This study aimed to investigate the role of MLH1 in the gastric cancer pathogenesis and to characterize MLH1-deficient cases compared with MLH1-preserved cases, focusing on tumor-related factors and patient background. We also aimed to identify the genes potentially associated with phenotypic differences.

**Methods:**

Patients who underwent gastrectomy for gastric cancer between June 2019 and December 2023 and had histopathological diagnoses indicating MLH1 expression were included in this study. Clinicopathological correlations were examined in 85 patients, including 14 MLH1-deficient and 71 MLH1-preserved cases. Finally, the gene characteristics of MLH1 deficiency were analyzed for correlations with MLH1 expression using cBioPortal.

**Results:**

The MLH1-deficient group showed significant tumor depth progression, reduced alcohol consumption, fewer *Helicobacter pylori* infections, and low lymphocyte percentages in preoperative blood tests. Aldehyde dehydrogenase 1A1, nuclear factor kappa B1, and programmed death-ligand 1 were identified as candidate genes associated with this phenotype.

**Conclusion:**

We observed differences in the gastric adenocarcinoma properties according to MLH1 expression and identified important candidate genes to elucidate the pathological mechanisms.

## Background

Gastric cancer is the fifth most common cancer worldwide in terms of incidence and the fifth leading cause of death. Therefore, improved treatment modalities are needed [1]. In addition to age, sex, and race, several dietary and lifestyle risk factors, such as salt-preserved foods, N-nitroso compound-containing foods, tobacco smoke, alcohol consumption, and obesity, have been demonstrated to contribute to the development of gastric cancer. The eradication of *Helicobacter pylori* (*H. pylori*) significantly reduces the incidence of gastric cancer [2].

The Cancer Genome Atlas (TCGA) has proposed that the subtypes of gastric cancer can be categorized further into Epstein–Barr virus (EBV)-positive, microsatellite instability (MSI), genomically stable (GS), and chromosomal instability (CIN). Providing treatment according to the type is considered an appropriate strategy [3]. Among these, the MSI type accounts for approximately 22% of all gastric cancer cases; however, its clinical and other properties remain unclear.

MSI, due to impaired base mismatch repair (MMR) during deoxyribonucleic acid (DNA) replication, is referred to as MSI-high, and gastric cancers with this condition are termed MSI-high gastric cancer. Corso et al. reported that MSI-high gastric cancer has a favorable prognosis, with the phenotype being associated with older age, female sex, antral tumor location, intestinal histotype, and less infiltration of the serosa. Moreover, lymph node involvement is rare and is limited to a few metastatic nodes [4]. Falchetti et al. reported a good prognosis with long-term overall survival and significant mutL homolog 1 (MLH1) deficiency [5].

In the MAGIC trial evaluating perioperative chemotherapy for gastric cancer, patients with MMR deficiency (MMRD or MSI-high status showed favorable prognosis when treated with surgery alone. However, patients treated with chemotherapy demonstrated worse outcomes. If independently validated, MSI or MMRD determined by preoperative biopsy may be used to select perioperative chemotherapy [6]. In the CheckMate 142 trial on colorectal cancer, nivolumab achieved durable responses and disease control in dMMR/MSI-high metastatic cancer, suggesting the effectiveness of immune checkpoint inhibitors in MSI-high gastric cancer [7].

In sporadic MSI-high gastric cancer, a decreased MLH1 expression due to acquired methylation of its promoter region is the primary cause of a decreased MMR function. Furthermore, abnormal MLH1 expression accounts for most cases of MSI-high gastric cancers [8]. MLH1 has been reported to correlate with CpG island methylation and the CpG island methylator phenotype. Additionally, the methylation status of MLH1 may be a prognostic biomarker for patients with gastric cancer [9].

*H. pylori* infection induces DNA methylation in the gastric mucosa and is associated with an increased risk of gastric cancer. High methylation of genes related to *H. pylori* infection is associated with CIMP positivity and MLH1 methylation in primary gastric cancer tissues [10].

Although MLH1-deficient gastric cancer is expected to exhibit unique characteristics, many unknown clinicopathological features and recommended treatments are still under investigation In particular, the background of the patient’s general condition and blood test results are critical to consider, as they may be indicators of the effectiveness of gastric cancer treatment. However, many uncertainties remain in this regard. Immune status indicators based on blood cell fractions, including the neutrophil-to-lymphocyte ratio (NLR), platelet-to-lymphocyte ratio (PLR), and lymphocyte-to-monocyte ratio (LMR), as well as nutritional indicators, such as the prognostic nutritional index (PNI) and C-reactive protein–albumin–lymphocyte (CALLY) index, are considered important in the prognosis and susceptibility of gastric cancer [11–14]. When examining the characteristics of MLH1-deficient gastric cancer, these indicators are essential and aid in selecting an appropriate treatment. However, many factors influencing causative gene expression changes remain unclear.

Based on the above information, this study aimed to clarify the characteristics of MLH1-deficient gastric cancer by comparing it with MLH1-proficient gastric cancer, focusing on tumor factors and patient backgrounds. We also attempted to identify any possible causative genes to reveal the underlying mechanism.

## Materials and methods

### Patients

Patients diagnosed with gastric adenocarcinoma between June 2019 and December 2023 who underwent gastric surgery at Chiba University Hospital were included in this study. Patients were eligible if they (1) were > 20 years old, (2) were pathologically diagnosed with gastric adenocarcinoma, (3) did not have multiple gastric cancers or any other malignant tumors in the stomach, (4) did not receive any radiation or chemotherapy before surgery, and (5) were evaluated for MLH1 expression. Data from 85 patients were retrospectively analyzed after excluding those who did not meet these criteria.

### Clinical specimens

We obtained specimens from these patients and evaluated MLH1 expression. The Japanese Classification of Gastric Carcinoma, 14th edition, was used to classify gastric cancer stages in these patients. MLH1 expression status was examined by immunostaining with Monoclonal Mouse Anti-Human MutL Protein Homolog 1 (Clone ES05 ready-to-use, DAKO Japan, Tokyo, Japan) using DAKO Autostainer Link 48 (Agilent, CA, USA) at the Pathology Department of Chiba University Hospital.

### Clinicopathological features

Age, sex, body mass index (BMI), alcohol consumption, smoking history, status of *H. pylori* infection, and history of diabetes mellitus (DM) and other malignant diseases were selected as patient factors. Alcohol consumption was assessed based on medical records and patient interviews. Patients were classified as having alcohol consumption if they reported daily alcohol intake or a history of heavy drinking, whereas those with only occasional social drinking or no alcohol consumption were classified as non-drinkers. Patients were classified as *H. pylori positive* if they had a current or past infection, including those with a history of eradication, and as *H. pylori negative* if they had no eradication history and a serum antibody level < 3 U/mL. Diabetes was defined as a previous diagnosis of DM or a high glycosylated hemoglobin level. Tumor factors, including pathological T factor, N factor, stage, tumor differentiation, tumor infiltrative pattern, lymphatic invasion, venous invasion, and tumor markers (carcinoembryonic antigen [CEA] and cancer antigen 19 − 9 [CA19-9]), were selected as tumor factors and determined based on the pathological diagnosis. The correlation between these factors and MLH1 expression in tumors was also investigated.

### Blood test analysis

The correlation between the results of blood tests performed at the patient’s first visit or before surgery and MLH1 expression in the tumor was investigated. White blood cell count (WBC, /µL), neutrophil (%), and lymphocyte percentages (%) within the WBC fraction and C-reactive protein levels (mg/dL) were utilized to assess inflammation. In addition, the general state of the body, including nutritional status, was assessed using hemoglobin levels (g/dL), platelet count (×10^3^/µL), total protein (g/dL), albumin (ALB, g/dL), total cholesterol (mg/dL), and estimated glomerular filtration rate (mL/min/1.73 m^2^). The NLR, PLR, and LMR were also determined. Differences between the MLH1-preserved and MLH1-deficient groups for each item were examined using Student’s *t-*test.

### Correlation of gene expression with MLH1

Genes potentially associated with the clinicopathological characteristics of MLH1-deficient gastric cancer, as well as genes with potential therapeutic relevance, were selected and analyzed for correlations with MLH1 expression using the open-access cBioPortal for Cancer Genomics (http://cbioportal.org). For this analysis, RNA sequencing data from the Stomach Adenocarcinoma cohort (TCGA, Firehose Legacy) were used to evaluate correlations between MLH1 and the candidate gene expression levels.

### Evaluation of NF-κB expression

The expression of NF-κB was assessed using immunohistochemistry with a polymer-based peroxidase detection system. After antigen retrieval, the sections were incubated with anti-human NF-κB p65 polyclonal antibody (ab7970; Abcam, Cambridge, UK) overnight at 4 °C. After washing, immunoreactivity was visualized using an HRP-labeled polymer anti-rabbit secondary antibody (EnVision™+ System-HRP, anti-rabbit; K4003; Dako, Tokyo, Japan) for 1 h at 37 °C. Staining intensity was scored as no staining (0 points), weak (1 point), moderate (2 points), or strong (3 points). The proportion of the stained cancer area was classified as 0% (0 points), 1–20% (1 point), > 20–50% (2 points), or > 50% (3 points). An overall immunohistochemical score was calculated by multiplying the staining intensity score by the proportion score. Tumors with an overall score of ≤ 1 were defined as negative, whereas those with a score of ≥ 2 were defined as positive.

### Statistical analysis

Correlations between clinicopathological features (age, sex, pathological tumor factors, tumor status, and recurrence), patient background (smoking and alcohol history, past medical history of DM, and *H. pylori* infection), and blood test items were evaluated using Student’s *t-*test and Fisher’s exact test. JMP Pro version 18 software program (SAS Institute Inc., Cary, NC, USA) was used for all statistical analyses. *P-values* < 0.050 were considered to be significant and *p* < 0.100 were defined to indicate a trend. Odds ratios (ORs) and 95% confidence intervals (CIs) were calculated based on likelihood ratio statistics.

## Results

### Relationship between the expression of MLH1 and the clinicopathological characteristics of patients with gastric cancer

**Fig. 1:**
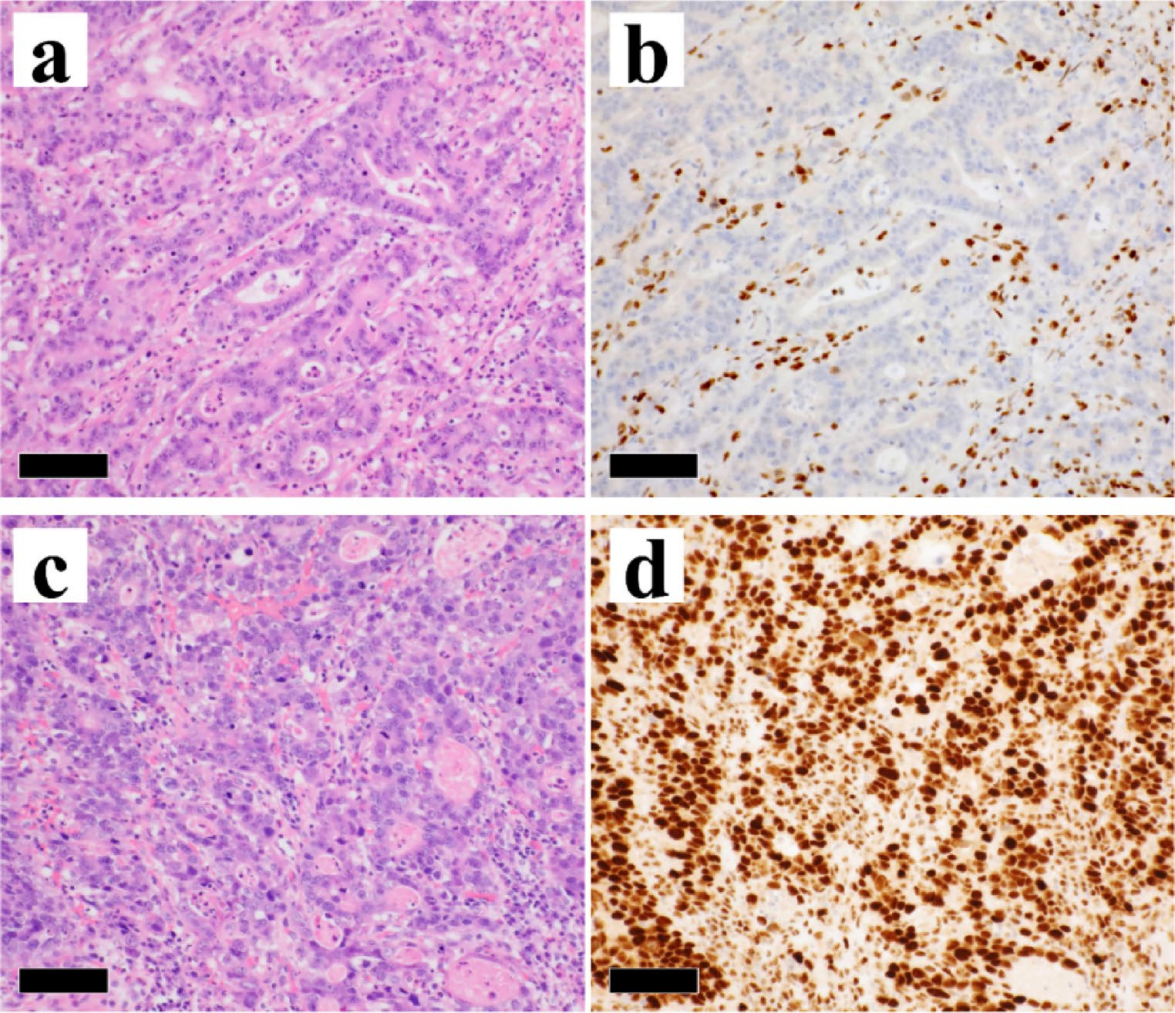
The immunohistochemical analysis of MLH1 expression in gastric cancer. **a-b**: MLH1-deficient case (a: H&E, b: MLH1), **c-d**: MLH1-proficient case (c: H&E, d: MLH1), Scale bar = 100µm.

The patient background and main tumor factors (age, sex, and pathological tumor factors) are presented in Table 1. Of the 85 patients, 71 (83.5%) were in the MLH1-preserved group and 14 (16.5%) were in the MLH1-deficient group (Fig. 1). Patient ages ranged from 40 to 93 years, with a median age of 76 years. Sixty (70.6%) patients were men, and 25 (29.4%) were women.

The pathological T factors for T1/T2/T3/T4 were 31/13/31/10, respectively; the pathological N factors for N0/N1/N2/N3 were 44/12/15/14, respectively; and 44 patients were negative (51.8%) for lymph node metastasis. Tumor differentiation included tub1/tub2 in 45 patients, pap in four, muc in one, por in 26, and sig in seven, with other types in two. In summary, differentiated type (tub1/tub2/pap) was noted in 49 patients and undifferentiated type (por/sig/muc) in 34 patients.

These clinicopathological features were compared between the MLH1-preserved group (preserved group) and the MLH1-deficient group (deficient group) (Table 2a). The age was significantly higher in the deficient group (73.5 ± 1.2 vs. 79.6 ± 1.9, *p* = 0.0103). An analysis of the tumor pathological factors demonstrated significant progression in the deficient group for tumor depth, with no significant differences in the LN metastasis or stage (pathological T factor, *p* = 0.0142; pathological N factor, *p* = 0.2412; stage *p* = 0.2406).

No significant differences were observed in tumor differentiation (*p* = 1.0000) or tumor infiltration patterns (*p* = 0.1190). However, lymphatic invasion demonstrated a trend toward progression (ly0 vs. ly1a/ly1b/ly1c, *p* = 0.0859), and venous invasion of tumors was significantly more common in the deficient group (v0 vs. v1a/v1b/v1c, *p* = 0.0282). No significant differences were identified in the tumor markers CEA and CA19-9 (CEA: *p* = 0.6824; CA19-9: *p* = 0.2595). Furthermore, no significant difference was observed in the recurrence rate (*p* = 0.4825). Body mass index (BMI), a possible indicator of nutritional status, was not significantly different between the two groups (*p* = 0.5459). A history of heavy alcohol consumption was less frequent in the MLH1-deficient group (*p* = 0.0028). Moreover, a history of *H. pylori* infection was significantly more common in the preserved group (*p* = 0.0294). No significant differences were identified in smoking history, DM, or history of other malignant diseases (smoking, *p* = 0.1890; DM, *p* = 0.3879; other malignant diseases, *p* = 1.0000).

To identify the independent factors associated with MLH1 deficiency, a multivariate logistic regression analysis was performed including age, pathological T factor, venous invasion, alcohol consumption, and Helicobacter pylori infection as covariates (Table 2b). The multivariate analysis demonstrated that alcohol consumption was independently associated with MLH1 deficiency, with heavy alcohol consumption being inversely associated with MLH1-deficient gastric cancer (odds ratio [OR] 0.12, 95% confidence interval [CI] 0.01–0.75, *p* = 0.0208). In contrast, age, pathological T factor, venous invasion, and H. pylori infection were not identified as independent factors associated with MLH1 deficiency.

### Correlation between MLH1 expression and blood test analysis

Blood test results at the initial visit and before surgery were compared between the MLH1-deficient and preserved groups (Table 3). The MLH1-deficient group had a significantly lower percentage of lymphocytes, which is a potential indicator of cancer immune status (*p* = 0.0324). The other parameters did not exhibit any clear correlations.

### Search for genes that correlate with MLH1 expression

Genes potentially associated with the clinicopathological characteristics of MLH1-deficient gastric cancer, including age, tumor invasion, alcohol consumption, and inflammatory/immune features, as well as potential therapeutic targets such as vascular endothelial growth factor receptor 2 (VEGFR2) and programmed death-ligand 1 (PD-L1), were selected, and correlations between MLH1 mRNA expression and the candidate gene expression levels were analyzed using cBioPortal (Table 4).

Regarding the older age observed in our MLH1-deficient cases, sirtuin genes were identified as targets. In the analysis with the cBioPortal database, sirtuin 6 (SIRT6) expression was significantly elevated in MLH1-deficient gastric cancer (*p* = 0.0022, Spearman’s correlation coefficient [ρ] = − 0.493).

Since our MLH1-deficient cases showed progressive tumor depth and increased vascular invasion, the vascular endothelial growth factor (VEGF) family, VEGFR2 (KDR), and C-X-C motif chemokine ligand 1 (*CXCL1*) genes were examined using the cBioPortal database. No correlation was detected between the VEGF family members (VEGF-A, VEGF-C, and VEGF-D) and MLH1. However, VEGFR2 demonstrated a trend toward reduced expression in MLH1-deficient gastric cancer (*p* = 0.0561, Spearman’s ρ = 0.321), whereas CXCL1 expression was significantly increased in MLH1-deficient tumors (*p* = 0.0481, Spearman’s ρ = − 0.332).

Because MLH1-deficient gastric cancer showed reduced alcohol consumption in the Chiba University case series, Aldehyde dehydrogenase 1 family, member A1 (ALDH1A1), and Aldehyde dehydrogenase 2 (ALDH2) were examined using the cBioPortal database. ALDH1A1 was significantly less expressed in MLH1-deficient gastric cancer (*p* = 0.0001, Spearman’s ρ = 0.596). In contrast, ALDH2 expression tended to be higher in MLH1-deficient gastric cancer (*p* = 0.0841, Spearman’s ρ = − 0.292).

Because of significant differences with the lymphocyte fraction and *H. pylori* infection in clinical 85 cases, we examined nuclear factor kappa (NF-κB) signaling and PD-L1 as immune- and inflammation-related genes in the cBioPortal database. An examination of NF-κB molecules revealed significantly high expression of NFKB1 in MLH1-deficient gastric cancer (*p* = 0.0003, Spearman’s ρ = − 0.563) and a trend toward higher expression of NFKB2 (*p* = 0.0501, Spearman’s ρ = − 0.329). Finally, examining PD-L1 (CD274), a marker of anti-programmed cell death protein 1 (PD-1)/PD-L1 therapy, PD-L1 expression was significantly high in MLH1-deficient gastric cancer (*p* = 0.0093, Spearman’s ρ = − 0.427).

### Association between the MLH1 status and NF-κB immunohistochemical expression

**Fig. 2:**
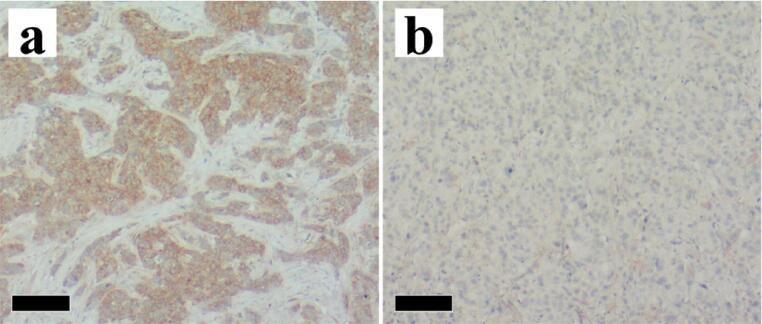
Cancer. **a**: NF-кВ positive case, **b**: NF-kB negative case, Scale bar = 100µm.

To validate the candidate genes suggested by the cBioPortal analysis, an immunohistochemical evaluation of NF-κB expression was performed (Fig. 2). NF-κB positivity was observed in 13 of 14 (92.9%) cases in the MLH1-deficient group, whereas it was observed in 35 of 71 (49.3%) cases in the MLH1-preserved group. NF-κB expression was significantly more frequent in MLH1-deficient gastric cancer than in MLH1-preserved gastric cancer (*p* = 0.0026) (Table 5).

## Discussion

In the present study, we investigated the clinicopathological characteristics of gastric cancer based on MLH1 expression. In terms of clinicopathological characteristics, patients in the MLH1-deficient group were older and had more advanced tumors than those in the preserved group. A trend toward more advanced vascular invasion was observed, although no significant differences were noted in the other stages of disease or recurrence. Regarding lifestyle habits, patients in the deficient group were significantly less likely to have a history of heavy alcohol consumption and fewer *H. pylori* infections. Additionally, pretreatment blood tests revealed significantly fewer lymphocytes in the white blood cell fraction in the deficient group.

Consistent with previous reports [4], the present study demonstrated that the MLH1-deficient group was older. In addition, patients with MLH1-deficient gastric cancer exhibited more advanced clinical T factors and greater lymphovascular invasion. Promoter methylation is crucial for the regulation of MLH1 expression and is likely influenced by inflammation; however, patients are less likely to have a history of heavy alcohol consumption or *H. pylori* infection, both of which can induce inflammation. Various factors, including the use of tobacco and alcohol consumption, are associated with a significant risk of gastric cancer [15], suggesting that the risk varies with the type of gastric cancer.

We examined the representative gene expression patterns corresponding to these clinical features in our 85 cases using the cBioPortal database and identified several promising genes.

**Table 1:** Patient characteristics (*n*=85)

Clinicopathological characteristics	Number of cases
Age median (min-max), years	76 (40–93)
Gender (male: female)	60: 25
Pathological T factor	
T1a/T1b	31
T2	13
T3	31
T4	10
Pathological N factor	
N0	44
N1	12
N2	15
N3	14
Stage	
IA/IB	39
IIA/IIB	19
IIIA/IIIB/IIIC	23
IV	4
Tumor differentiation	
tub1/tub2	45
pap	4
muc	1
por	26
sig	7
other	2
MLH1 expression	
Preserved	71
Deficient	14

MLH1, mutL homolog 1

Sirtuin family members play important roles in maintaining genome stability under stress, and sirtuin1 (SIRT1) regulates DNA damage signaling [16]. SIRT1 is also known as a dual player, promoter, and suppressor in gastric cancers [17]. In this study, SIRT6 was highly expressed in MLH1-deficient gastric cancers. Because SIRT6 upregulation suppresses SIRT1 [18], SIRT1 and SIRT6 may play important roles in gastric tumorigenesis.

**Table 2:** Association among the MLH1 status, clinicopathological characteristics and patient background. (a) Clinicopathological characteristics and patient background according to the MLH1 expression status. (b) A multivariate logistic regression analysis for the factors associated with MLH1 deficiency

Characteristics	MLH1 expression		*p*-value
	Preserved (*n* = 71)	Deficient (*n* = 14)	
Age	73.5±1.2 (76)	79.6±1.9 (79.5)	0.0103 *
Gender			
Male	52	8	0.3348
Female	19	6	
Tumor depth			
T1	30	1	0.0142 *
T2/T3/T4	41	13	
LN metastasis			
N0	39	5	0.2412
N1/N2/N3	32	9	
Stage			
I	35	4	0.2406
II/III/IV	36	10	
Tumor differentiation			
tub/pap	41	8	1.0000
por/sig/muc	28	6	
Tumor infiltrative pattern			
INFa/INFb	32	11	0.1190
INFc	21	2	
Lymphatic invasion			
ly0	39	4	0.0859
ly1a/ly1b/ly1c	32	10	
Venous invasion			
v0	28	1	0.0282 *
v1a/v1b/v1c	43	13	
CEA			
high value	10	1	0.6824
low value	61	13	
CA19-9			
high value	11	4	0.2595
low value	60	10	
Postoperative recurrence			
No	57	10	0.4825
Yes	14	4	
BMI	23.2±0.4	23.1±1.4	0.5459
Alcohol			
None or social only	35	13	0.0028 * *
Heavy consumption	35	1	
Smoking			
never	17	6	0.1890
current or ex	54	8	
*H. pylori* infection			
No	7	5	0.0294 *
Current or past	60	7	
Diabetes			
No	41	10	0.3879
Yes	30	4	
Other malignant disease			
No	41	8	1.0000
Yes	30	6	

Variables	Odds ratio	95% confidence interval	*p-value*
Age (per year)	1.05	0.96–1.17	0.2877
Pathological T factor (T2–4 vs. T1)	6.28	0.64–170.54	0.1229
Venous invasion (present vs. absent)	2.01	0.17–53.10	0.5991
Alcohol consumption (heavy vs. none/social)	0.12	0.01–0.75	0.0208 *
Helicobacter pylori infection (present vs. absent)	0.67	0.13–3.50	0.6288

CEA, carcinoembryonic antigen; CA19-9, cancer antigen 19-9; INF, infiltrative growth pattern; MLH1, mutL homolog 1; BMI, body mass index (kg/m^2^); *H. pylori*, *Helicobacter pylori*

Age data are presented as the mean ± standard error of the mean (median). BMI Data are displayed as the mean ± standard error of the mean

Statistical significance was defined as follows: *p < 0.05, **p < 0.01, ***p < 0.001

Alcohol-degrading enzyme ALDH1 is highly expressed in MLH1-proficient gastric cancers and responds to high alcohol intake. Moreover, ALDH1 is a stem cell marker. We have previously reported ALDH1-induced treatment resistance in esophageal squamous cell carcinoma [19]. Furthermore, ALDH1 is elevated in *H. pylori* infection [20]; therefore, both *H. pylori* infection and alcohol consumption are associated with elevated ALDH1 expression. High ALDH1 expression has been reported to be associated with a poor prognosis in cancer [21], whereas a low ALDH1 expression in MSI-high gastric cancer may be one of the factors contributing to a favorable prognosis.

**Table 3:** Relation between the

Items	MLH1 expression		*p*-value
	Preserved	Deficient	
WBC	6235.2±191.9	6128.6±351.7	0.7926
Neutrophil (%)	63.96±1.01	67.22±2.69	0.2727
Eosinophil (%)	2.71±0.30	3.85±1.07	0.3245
Basophil (%)	0.71±0.04	0.66±0.10	0.6913
Monocyte (%)	6.19±0.20	6.52±0.37	0.4409
Lymphocyte (%)	26.42±0.94	21.53±1.91	0.0324*
HGB	12.29±0.24	11.73±0.81	0.5200
PLT	230.4±9.7	247.4±20.2	0.4580
TP	7.00±0.06	6.96±0.23	0.8699
ALB	4.00±0.05	3.91±0.22	0.7007
eGFR	61.35±2.56	66.18±4.35	0.3491
T-CHO	184.5±4.2	187.1±12.4	0.8407
CRP	0.370±0.117	0.394±0.180	0.9141
NLR	2.799±0.170	3.614±0.461	0.1157
PLR	155.9±8.4	219.4±30.3	0.0616
LMR	4.59±0.23	3.58±0.48	0.0701

MLH1, mutL homolog 1; WBC, white blood cell count (/μL); HGB, hemoglobin (g/dL); PLT, platelet count (×10^3^/μL); TP, total protein (g/dL); ALB, albumin (g/dL); eGFR, estimated glomerular filtration rate (mL/min/1.73 m^2^); T-CHO, Total cholesterol (mg/dL); CRP, C-reactive protein (mg/dL); NLR, neutrophil-to-lymphocyte ratio, PLR, platelet-to-lymphocyte ratio; LMR, lymphocyte-to-monocyte ratio. Data are presented as the mean ± standard error of the mean

Statistical significance was defined as follows: *p < 0.05, **p < 0.01, ***p < 0.001

MLH1-deficient gastric cancer demonstrated severe vascular invasion, but exhibited a trend toward a reduced VEGFR2 expression. VEGFR2 is important in angiogenesis [22] and is the target of ramucirumab [23]. Currently, no reports have elucidated an association between ramucirumab and MLH1 in gastric cancer, and this remains an area of interest. *CXCL1* is another gene that enhances angiogenesis and venous invasion [24]. CXCL1 was also significantly upregulated in MLH1-deficient gastric cancer, which may explain the progressive T factor and high degree of vascular invasion. Thus, CXCL1 is a promising therapeutic target for MLH1-deficient gastric cancer.

**Table 4:** The candidate genes related to the MLH1 deficient status

Clinicopathological features in MLH1 deficient gastric cancer	Candidate genes	*p*-value	Spearman	Relative mRNA expression	Footnote	References
Elder	Sirtuin 6	0.0022**	-0.493	high	Sirt6 upregulation suppresses Sirt1, important regulator in DNA damage stress.	[16–18]
Higher invasion	VEGF family	n.s.	-	not related	-	
	VEGFR2 (KDR)	0.0561	0.321	low	VEGF receptor 2 / target of ramucirumab	[22, 23]
	CXCL1	0.0481*	-0.332	high	Increases angiogenesis and venous infiltration	[24]
Reduced alcoholic consumption	ALDH1A1	0.0001***	0.596	low	Alcohol degrading enzyme and cancer stem cell marker. The expression is also elevated by *H.pylori* infection.	[19–21]
	ALDH2	0.0841	-0.292	high	Alcohol degrading enzyme	
Inflammation	NFKB1	0.0003***	-0.563	high	Transcriptional regulators of inflammation and immune-related gene expression	[25]
	NFKB2	0.0501	-0.329	high		
Therapeutic target	PD-L1 (CD274)	0.0093**	-0.427	high	target of anti-PD-1/PD-L1 therapy	[26]

PD-L1, programmed death ligand 1; mRNA, messenger ribonucleic acid; MLH1, mutL homolog 1; VEGF, vascular endothelial growth factor; VEGFR2, vascular endothelial growth factor receptor 2; CXCL1, C-X-C motif chemokine ligand 1; ALDH1A1, aldehyde dehydrogenase 1 family, member A1; ALDH2, aldehyde dehydrogenase 2; NFKB1, nuclear factor kappa B subunit 1; NFKB2, nuclear factor kappa B subunit 2; PD-L1, Programmed cell Death ligand 1

Statistical significance was defined as follows: *p < 0.05, **p < 0.01, ***p < 0.001

**Table 5:** Association between the MLH1 status and NF-κB immunohistochemical expression

		NF-κB expression			*p* value
		Positive (*n* = 48)	Negative (*n* = 37)	Positive rate	
**MLH1**	Preserved (*n* = 71)	35	36	49.3%	0.0026**
**expression**	Deficient (*n* = 14)	13	1	92.9%	

MLH1, MutL homolog 1; NF-κB, nuclear factor kappa B

Statistical significance was defined as follows: *p < 0.05, **p < 0.01, ***p < 0.001

NF-κB signaling is known as a master gene for inflammation and immunity, and we found that NF-κB expression was elevated in MLH1-deficient gastric cancer with IHC in this study. To the best of our knowledge, this is one of the first studies to demonstrate a clear association between MLH1 deficiency and enhanced NF-κB activity in gastric cancer using transcriptomic data and immunohistochemistry. Although we have reported on NF-κB expression in carcinomas of peritoneal dissemination [25], NF-κB signaling may significantly affect the nature of MLH1-deficient gastric cancer and their response to therapies, such as cancer immunotherapy.

Immunotherapy targeting PD-1/PD-L1 is a promising therapy for gastric cancer. Additionally, anti-PD-1-based therapy has been reported to be highly effective in MSI-high gastric cancer [26], with a high PD-L1 expression in MLH1-deficient gastric cancer in our study. These findings are consistent with and support the results of a previous report. Given that MLH1/MMR deficiency may include hereditary subsets, careful molecular stratification (sporadic vs. Lynch/Lynch-like) is warranted, and additional somatic alterations reported in MSI-high colorectal cancer (e.g., KRAS, ITGB3BP, CLEC16A, ARHGEF28, PIK3CA, and RBM26) may also be candidates to explore in MLH1-deficient gastric cancer [27].

The limitations of this study include the fact that it was performed at a single institution with only 85 cases, a relatively small sample size, and the fact that some cases were recently operated on, limiting the time available to assess the prognosis. An additional limitation is the difference between our 85 cases and the cBioPortal dataset. It remains to be determined whether the Chiba University cases we studied show the same correlation of RNA expression as in the cBioPortal database. Therefore, the genes that could explain the MLH1-deficient gastric cancer characteristics presented in this study are still in the hypothetical stage. Therefore, further studies are warranted in the future.

## Conclusion

We identified differences in the gastric cancer properties attributed to MLH1 expression and identified several important candidate genes associated with MLH1 expression, including *ALDH1A1*, *CXCL1*, *NFKB1*, and *PD-L1*. This study will therefore help elucidate the pathological mechanisms underlying MLH1-deficient gastric cancer.

## References

[1] Bray F, et al. Global cancer statistics 2022: GLOBOCAN estimates of incidence and mortality worldwide for 36 cancers in 185 countries. CA Cancer J Clin. 2024;74(3):229–63. 38572751 10.3322/caac.21834

[2] Grantham T, et al. Epidemiology of Gastric Cancer: Global Trends, Risk Factors and Premalignant Conditions. J Community Hosp Intern Med Perspect. 2023;13(6):100–6. 38596548 10.55729/2000-9666.1252 PMC11000854

[3] Cancer Genome Atlas Research Network. Comprehensive molecular characterization of gastric adenocarcinoma. Nature. 2014;513(7517):202-9. 10.1038/nature13480 PMC4170219 25079317

[4] Corso G, et al. Correlation of microsatellite instability at multiple loci with long-term survival in advanced gastric carcinoma. Arch Surg. 2009;144(8):722–7. 19687375 10.1001/archsurg.2009.42

[5] Falchetti M, et al. Gastric cancer with high-level microsatellite instability: target gene mutations, clinicopathologic features, and long-term survival. Hum Pathol. 2008;39(6):925–32. 18440592 10.1016/j.humpath.2007.10.024

[6] Smyth EC, et al. Mismatch Repair Deficiency, Microsatellite Instability, and Survival: An Exploratory Analysis of the Medical Research Council Adjuvant Gastric Infusional Chemotherapy (MAGIC) Trial. JAMA Oncol. 2017;3(9):1197–203. 28241187 10.1001/jamaoncol.2016.6762 PMC5824280

[7] Overman MJ, et al. Nivolumab in patients with metastatic DNA mismatch repair-deficient or microsatellite instability-high colorectal cancer (CheckMate 142): an open-label, multicentre, phase 2 study. Lancet Oncol. 2017;18(9):1182–91. 28734759 10.1016/S1470-2045(17)30422-9 PMC6207072

[8] Leite M, et al. MSI phenotype and MMR alterations in familial and sporadic gastric cancer. Int J Cancer. 2011;128(7):1606–13. 20533283 10.1002/ijc.25495

[9] Shigeyasu K, et al. Clinical Significance of MLH1 Methylation and CpG Island Methylator Phenotype as Prognostic Markers in Patients with Gastric Cancer. PLoS ONE. 2015;10(6):e0130409. 26121593 10.1371/journal.pone.0130409 PMC4488282

[10] Tahara S, et al. Helicobacter pylori infection associated DNA methylation in primary gastric cancer significantly correlates with specific molecular and clinicopathological features. Mol Carcinog. 2024;63(2):266–74. 37846801 10.1002/mc.23650

[11] Tomás TC, et al. Neutrophile-to-lymphocyte, lymphocyte-to-monocyte, and platelet-to-lymphocyte ratios as prognostic and response biomarkers for resectable locally advanced gastric cancer. World J Gastrointest Oncol. 2022;14(7):1307–23. 36051098 10.4251/wjgo.v14.i7.1307 PMC9305575

[12] Pan YC, et al. Preoperative lymphocyte-to-monocyte ratio (LMR) could independently predict overall survival of resectable gastric cancer patients. Med (Baltim). 2018;97(52):e13896. 10.1097/MD.0000000000013896 PMC6314713 30593200

[13] Song S, et al. Derived neutrophil to lymphocyte ratio and monocyte to lymphocyte ratio may be better biomarkers for predicting overall survival of patients with advanced gastric cancer. Onco Targets Ther. 2017;10:3145–54. 28706446 10.2147/OTT.S138039 PMC5495088

[14] Fukushima N, et al. Prognostic significance of the preoperative C-reactive protein-albumin-lymphocyte (CALLY) index on outcomes after gastrectomy for gastric cancer. Surg Today. 2024;54(8):943–52. 38491233 10.1007/s00595-024-02813-1

[15] Ko KP. Risk Factors of Gastric Cancer and Lifestyle Modification for Prevention. J Gastric Cancer. 2024;24(1):99–107. 38225769 10.5230/jgc.2024.24.e10 PMC10774756

[16] Rasti G, et al. SIRT1 regulates DNA damage signaling through the PP4 phosphatase complex. Nucleic Acids Res. 2023;51(13):6754–69. 37309898 10.1093/nar/gkad504 PMC10359614

[17] Poniewierska-Baran A, Warias P, Zgutka K. Sirtuins (SIRTs) As a Novel Target in Gastric Cancer. Int J Mol Sci, 2022. 23(23):15119. 10.3390/ijms232315119 PMC9737976 36499440

[18] Seo JH, et al. Sirt6-Mediated Cell Death Associated with Sirt1 Suppression in Gastric Cancer. Cancers (Basel). 2024;16(2):387. 38254877 10.3390/cancers16020387 PMC10814469

[19] Qian L, et al. An anti-alcoholism drug, disulfiram and copper complex improves radio-resistance of tumor-initiating cells in esophageal squamous cell carcinoma. Esophagus. 2023;20(1):134–42. 36121574 10.1007/s10388-022-00948-z

[20] Levi E, et al. Cancer stem cells in Helicobacter pylori infection and aging: Implications for gastric carcinogenesis. World J Gastrointest Pathophysiol. 2014;5(3):366–72. 25133037 10.4291/wjgp.v5.i3.366 PMC4133534

[21] Li XS, et al. ALDH1A1 overexpression is associated with the progression and prognosis in gastric cancer. BMC Cancer. 2014;14:705. 25253129 10.1186/1471-2407-14-705 PMC4180139

[22] Hironaka S. Anti-angiogenic therapies for gastric cancer. Asia Pac J Clin Oncol. 2019;15(4):208–17. 31111678 10.1111/ajco.13174

[23] Iwasa S, et al. The clinical position of ramucirumab-containing regimens for advanced gastric cancer: a review of clinical trial data. Future Oncol. 2022;18(21):2709–21. 35703103 10.2217/fon-2022-0281

[24] Kasashima H, et al. Clinicopathologic significance of the CXCL1-CXCR2 axis in the tumor microenvironment of gastric carcinoma. PLoS ONE. 2017;12(6):e0178635. 28575019 10.1371/journal.pone.0178635 PMC5456266

[25] Sekino N, et al. The Antitumor Effects of Metformin on Gastric Cancer In Vitro and on Peritoneal Metastasis. Anticancer Res. 2018;38(11):6263–9. 30396946 10.21873/anticanres.12982

[26] Pietrantonio F, et al. Predictive role of microsatellite instability for PD-1 blockade in patients with advanced gastric cancer: a meta-analysis of randomized clinical trials. ESMO Open. 2021;6(1):100036. 33460964 10.1016/j.esmoop.2020.100036 PMC7815473

[27] Ofuchi T et al. Identification of the Lynch syndrome and Lynch-like syndrome specific somatic mutations in microsatellite instability-high colorectal cancer cases. Surg Today, 2025. 10.1007/s00595-025-03212-w 10.1007/s00595-025-03212-w 41441884

